# Research on the Effect of Sodium Aluminate on the Early Performance Enhancement and Mechanism of Phosphogypsum-Based Cementitious Materials

**DOI:** 10.3390/ma18122707

**Published:** 2025-06-09

**Authors:** Xiaoming Liu, Shuchao Zhai, Xihe Zhang

**Affiliations:** 1 School of Civil Engineering, Central South University, Changsha 410075, China; 207076@csu.edu.cn (X.L.); 234811149@csu.edu.cn (S.Z.); 2 Power Construction Corporation of China Guiyang Engineering Corporation Limited, Guiyang 550081, China

**Keywords:** phosphogypsum, sodium aluminate, early performance, ettringite, microstructure, mechanism

## Abstract

Phosphogypsum (PG) is used to prepare eco-friendly cementitious materials, representing a high-value resource utilization approach. However, there are some shortcomings, such as a long setting time and low early strength in phosphogypsum-based cementitious materials (PBCMs), which limit their engineering applications. This work aimed to improve their early performance by adding sodium aluminate. In particular, the effects on the compressive strength, setting time, and fluidity of PBCMs were investigated. Additionally, the effect of sodium aluminate on hydration was analyzed by X-ray diffraction (XRD) and scanning electron microscopy (SEM). The results indicate that the addition of sodium aluminate results in a significant enhancement in 3 d compressive strength and an obvious procoagulant effect on setting time in PBCMs. When the content of sodium aluminate reaches 1 wt.%, the 3 d compressive strength of PBCMs can reach 10.72 MPa. Compared with the control group (A0, without sodium aluminate), the 3 d compressive strength is improved by 587.39%, and the final setting time is shortened by 4 h 4 min. The microscopic test results show that sodium aluminate can provide sufficient aluminum components at the early stage of hydration, which could effectively enable more phosphogypsum to participate in hydration and accelerate the early part of the process of the hydration reaction. This is conducive to increasing the number of early hydration products of ettringite (AFt) and C-A-S-H gel to improve the early compressive strength and shorten the setting time.

## 1. Introduction

Phosphogypsum (PG) is an industrial waste generated during the industrial production of phosphoric acid and is one of the major solid wastes in China [1]. Phosphogypsum is emitted at a rate of more than 200 million tons per year worldwide [2,3]. To date, the cumulative stockpile has reached more than 3 billion tons worldwide [4], but the comprehensive utilization rate of phosphogypsum is only about 25% in China [5,6]. The main way to handle phosphogypsum is to store it in open-air stockpiles above ground level, which not only places great pressure on the ecological environment but also wastes a large amount of valuable land resources [7,8]. At the same time, the heavy metal ions in phosphogypsum also pollute groundwater resources [9,10]. Therefore, improving the utilization of phosphogypsum is an effective way of eliminating phosphogypsum and reducing the environment pollution it causes.

The main component of PG is gypsum (CaSO_4_·2H_2_O), which is an air-hard cementitious material [11]. PG, as the main raw material, is used to prepare green cementitious materials, which could eliminate a large amount of PG. Unlike Portland cement production, requiring “two-stage grinding and one-stage calcination”, phosphogypsum-based cementitious materials (PBCMs) can be prepared without such energy-intensive processes, which could significantly reduce CO_2_ emissions and the consumption of energy and resources [12]. In recent years, the preparation of PBCMs has attracted increased attention among scholars. Huang et al. [13] prepared phosphogypsum-based calcination-free cement with phosphogypsum, steel slag, ground granulated blast-furnace slag, and limestone. Amrani et al. [14] focused on the stabilization of PG by stabilizers, such as clayey soil, fly ash, lime, and calcareous material.

PBCMs have a high strength in later age. Lam et al. [15] prepared phosphogypsum–slag cement with phosphogypsum, slag, cement, and quicklime, and the 28 d paste compressive strength could reach 40 MPa. Chen et al. [16] prepared foamed phosphogypsum-based composite cementitious materials with hemihydrate phosphogypsum, fly ash, and lime, and their 28 d compressive strength could reach 14.08 MPa. Liu et al. [17,18] prepared a phosphogypsum-based all-solid-waste geopolymer with phosphogypsum, recycled fined powder, and ground granulated blast-furnace slag by using sodium hydroxide and water-glass as alkalinity activators, and its 28 d compressive strength could exceed 30 MPa. Additionally, PBCMs exhibit excellent durability, which affects their service life [19]. Zhang et al. [20] found that PG could inhibit material shrinkage. This is because of the slight expansive behavior of gypsum during the early stage, improving long-term performance [21,22]. Pinto et al. [23] found that PBCMs exhibited a good sulfate resistance. Pratap et al. [24] worked on the impact of varying PG contents on the chloride resistance and indicated that the mix exhibited the lowest chloride penetration when the content of PG was 30%.

As discussed above, PBCMs have many advantages, but there are some disadvantages, such as longer setting times and lower early strength. Some studies [1,20] have indicated that PG typically results in a long setting time in alkali-activated materials. Xu et al. [10] elucidated that the strength development of PBCMs primarily relied on hydration products formed through the reaction between GGBS and gypsum. But in the early stage, the slow dissolution rate of the active components in the GGBS was not conducive to forming enough hydration products to develop strength [25]. Wang et al. [26] pointed out that the early strength was mainly provided by ettringite, which connected adjacent phosphogypsum particles to form a three-dimensional reticulate structure. The pores were filled gradually by C-S-H gel, resulting in a densified solid matrix, along with the process of hydration to promote the development of strength.

In PBCMs, a large amount of phosphogypsum can provide sufficient sulfate ions and calcium ions. The dissolution of the aluminum component is a key step for the generation of ettringite, which is also a key factor in the formation of strength in the early stage. To solve this problem, some scholars have improved the early strength by steam conservation [27,28,29]. Dong et al. [21] found it could shorten the setting time by adding sulfoaluminate cements. Lin et al. [30] found that sodium hydroxide could significantly shorten the setting time, but it was not conducive to the later stage of strength development. The essence of the above measures to improve early performance is to enhance the participation of aluminates in the early hydration reaction.

Sodium aluminate is the main component of liquid alkaline accelerators used in ordinary Portland cement systems. Yang et al. [31] confirmed that sodium aluminate could accelerate the formation of ettringite. It generates tetrahydroxy aluminate ions and hydroxide ions when dissolved in water, providing an abundant source of aluminum species that facilitates the early hydration of cementitious materials [32,33]. These ions can react with sufficient calcium and sulfate ions to form ettringite, while the hydroxide ions produced create an alkaline environment that further promotes the hydrolysis and dissolution of slag [34].

**Figure 1:**
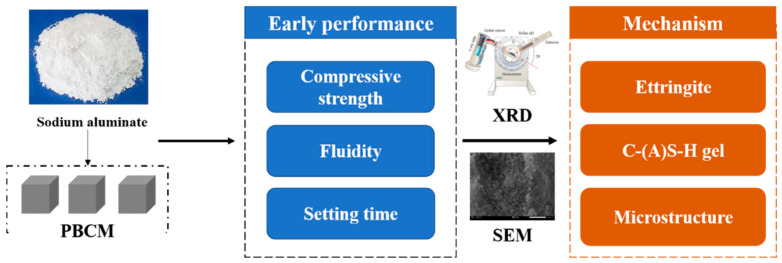
Workflow diagram of this work.

As shown in Figure 1, the research work aims to solve the problem of the setting time and the early strength of PBCMs by adding sodium aluminate. The influence of sodium aluminate on the early performance and the hydration mechanism in PBCMs was investigated. XRD and SEM were used to analyze the influence of sodium aluminate on the compressive strength, setting time, fluidity, hydration product phase composition, and microscopic morphology of PBCMs. This work has a positive significance for the large-scale use of PBCMs and the resource utilization of PG, which helps to reduce the environment pollution and the construction cost.

## 2. Materials and Methods

### 2.1. Materials

**Figure 2:**
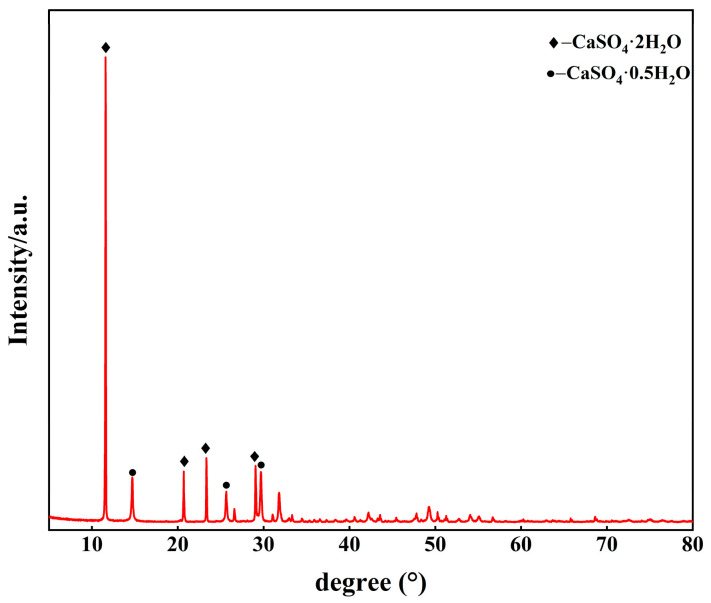
Mineral phases of PG and GGBS.

The PG used in the study was supplied by Guizhou Wengfu Phosphorus Mining Group Co., Ltd. (Guizhou, China), with a moisture content of 6.19%. Ground granulated blast-furnace slag (GGBS) was of S95 grade, with a specific surface area of more than 420 m^2^/kg and a density of 2.7 g/cm^3^. The mineral phases of PG and GGBS are given in Figure 2. The calcium oxide content in the quicklime was more than 95.26%. The main chemical composition of the raw materials is shown in Table 1. Sodium aluminate was an analytically pure reagent. A polycarboxylate ether (PCE)-based superplasticizer (Type 556P, Sika Construction Materials Co., Ltd., Suzhou, China) was employed in this study.

### 2.2. Mix Proportion Design and Specimen Preparation

There are a few phosphorus and fluorine impurities in PG, requiring pretreatment for their removal. Firstly, PG was mixed with quicklime at a mass ratio of 90:1, along with 0.55 times the mass of phosphogypsum in water. The mixture was put into a mixer for 6 min (3 min for slow mixing followed by 3 min for fast mixing), and stored for 24 h at room temperature before use. The detailed experimental mix proportion is shown in Table 2. Sodium aluminate was dissolved in water and then poured into the mixer. It was first mixed slowly for 2 min and stirred quickly for 2 min to make sure that the slurry was well mixed. And then, the stirred slurry was poured into the mold (40 mm × 40 mm × 40 mm). The samples were cured in the standard curing chamber (20 ± 3 °C, more than 95% relative humidity). After demolding, they were transferred back to the curing chamber and maintained until the specified testing age. 

### 2.3. Physical and Mechanical Properties Test

Strength tests on the specimens cured for 3 d, 7 d, and 28 d were carried out by the Constant Loading Automatic Pressure Testing Machine TSY-2000A (Shanghai Luda Machinery & Instrument Co., Ltd., Shanghai, China) with a loading speed of 2.4 KN/S.

The determination of the setting time followed the standard method outlined in GB/T 1346-2011 [35].

The fluidity of PBCM was determined according to GB/T 8077-2000 [36].

### 2.4. Microscopic Test

Respectively, samples A0, A2, and A4 at a specified age were first crushed into small pieces and immersed in anhydrous ethanol for 7 d to terminate the hydration. The pieces were put into a vacuum-drying oven to dry for 24 h.

A fragmented sample (less than 1 cm in thickness and diameter) with a relatively intact surface micro-morphology was selected, and the specimen was subjected to the SEM test after gold spraying.

The rest of the fragmented samples were ground into a powder using a mortar and pestle for the XRD test. Phase analysis was performed using a Bruker D8 Advance X-ray diffractometer (Bruker (Beijing) Technology Co., Ltd., Beijing, China). The scanning speed was set to 2°/min, and the scanning angle range was from 5° to 80°.

## 3. Results and Discussion

### 3.1. Compressive Strength

**Figure 3:**
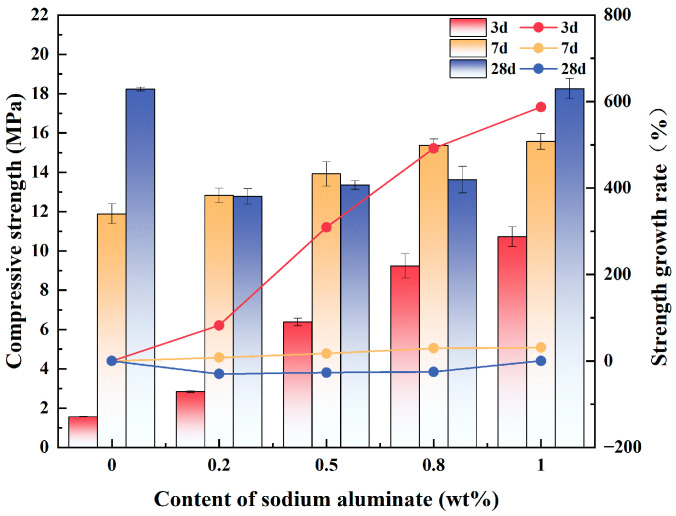
Effect of varying content of sodium aluminate on the compressive strength of PBCMs.

Figure 3 demonstrates the effect of varying the content of sodium aluminate on the compressive strength of PBCMs at different ages. The 3 d compressive strength of PBCMs exhibited a progressive increase with increasing sodium aluminate content. And the addition of sodium aluminate could significantly improve the 3 d compressive strength of PBCMs. Comparatively, the 3 d compressive strength of A0 was only 1.75 MPa, while that of A3 could reach a maximum of 10.72 MPa. Compared with the A0 group, the 3 d compressive strength of specimens after adding 0.2 wt.%, 0.5 wt.%, 0.8 wt.%, and 1 wt.% sodium aluminate increased by 82.26%, 309.19%, 491.88%, and 587.39%, respectively. Former research findings also demonstrated that sodium aluminate could substantially improve the early-stage compressive strength of cementitious systems [31,34,37].

As the curing age increases, more GGBS participates in the hydration process, generating more hydration products. Consequently, the accelerating effect of sodium aluminate on hydration diminishes over time, resulting in a progressively reduced contribution to strength enhancement. For example, at a sodium aluminate content of 1 wt.%, the 7 d compressive strength reaches 15.57 MPa, representing a 39.66% increase relative to the A0 group (11.88 MPa). However, the compressive strengths of specimens A1 (12.77 MPa), A2 (13.35 MPa), and A3 (13.62 MPa) at 28 d are lower than that of the A0 group (18.23 MPa), which exhibits a decline relative to their respective 7 d strengths. Notably, the 28 d compressive strength of A3 reaches 18.25 MPa, which is nearly identical to that of the A0 group.

### 3.2. Fluidity

**Figure 4:**
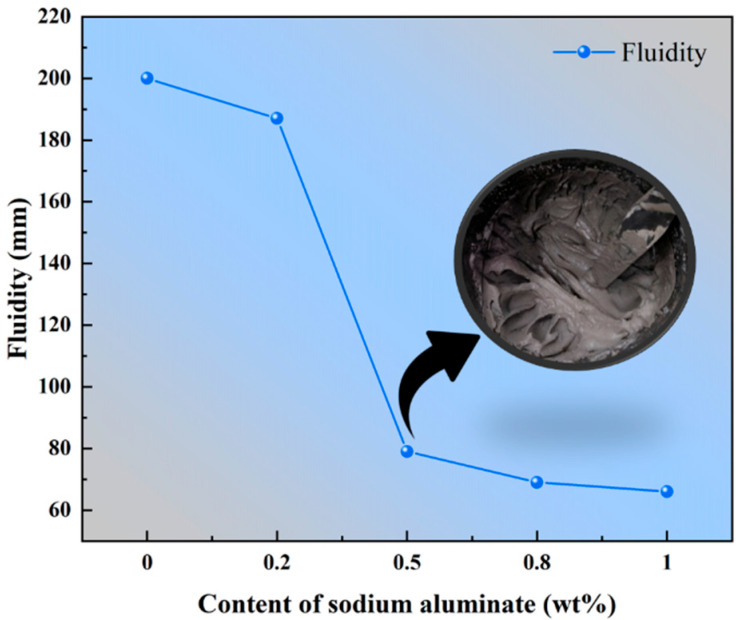
Effect of varying content of sodium aluminate on the fluidity of PBCMs.

The relationship between the slurry fluidity of PBCMs and the content of sodium aluminate is illustrated in Figure 4. As the sodium aluminate content increases, the fluidity variation trend of PBCM slurry exhibits a clear decrease. When the content increases form 0 wt.% to 0.2 wt.%, it can be seen that the fluidity drops from 200 mm to 187 mm. A further increase to 0.5 wt.% results in a substantial reduction, with the fluidity decreasing sharply to only 79 mm. Complete loss of fluidity is observed at 0.8 wt.% sodium aluminate content. Peer-reviewed studies conducted by some research groups have consistently supported this experiment result [38,39,40].

### 3.3. Setting Time

**Figure 5:**
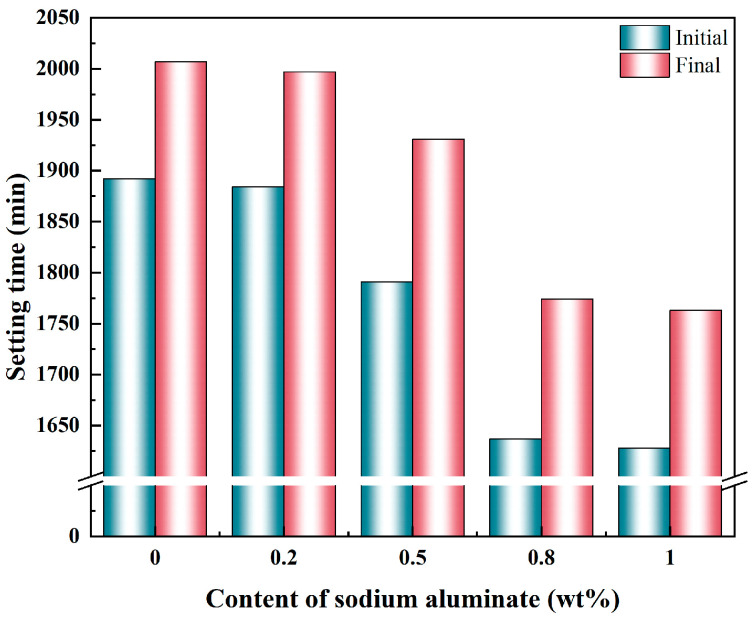
Effect of varying the content of sodium aluminate on the setting time of PBCMs.

The influence of sodium aluminate content on the setting time of PBCMs is illustrated in Figure 5. Sodium aluminate exerts a significant influence on the setting time of PBCMs. In the control group without sodium aluminate, the initial and final setting times were 1892 min and 2007 min, respectively. Both the initial and final setting times are markedly shortened by the addition of sodium aluminate, and the effect becomes more pronounced at higher contents. At 0.8 wt.% and 1 wt.% sodium aluminate, the initial setting times are reduced to 1637 min and 1628 min, while the final setting time is decreased to 1774 min and 1763 min. These reductions correspond to approximately 3 h 53 min and 4 h 4 min shorter than the control group’s final setting time. The above experimental results demonstrate that the addition of sodium aluminate significantly shortens the setting time of PBCMs. When the content exceeds 0.8 wt.%, the setting time exhibits little variation, indicating that 0.8 wt.% is the optimal content for accelerating the setting of PBCMs.

### 3.4. XRD Analysis

**Figure 6:**
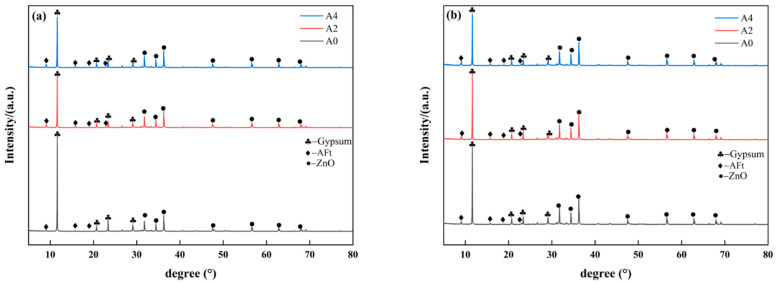
XRD patterns of PBCMs with different sodium aluminate contents at different ages: (**a**) 3 d; (**b**) 28 d.

The phase composition analysis of the hydration products in specimens containing different sodium aluminate contents at 3 d and 28 d is shown in Figure 6. As shown in Figure 6a, the control group (A0) exhibits relatively weak ettringite (AFt) diffraction peaks, indicating limited formation of ettringite during the early stage. It is one of the reasons for the extremely low strength of group A0 at 3 d.

Compared with the control group, the intensity of the gypsum peaks in the A2 and A4 groups is significantly reduced. In contrast, the intensity of the ettringite peak increases substantially along with the increase sodium aluminate content. This indicates that the participation of phosphogypsum in hydration and the formation of hydration products increases as sodium aluminate content increases. As the hydration reaction progresses from 3 d to 28 d, the intensity of the gypsum diffraction peaks in the A0, A2, and A4 sample groups further decreases as shown in Figure 6b. This indicates that phosphogypsum continued participating in the hydration process. In the meantime, the intensity of the ettringite diffraction peaks increases, suggesting that the amount of ettringite generated also rises, which correlates with the observed compressive strength development.

**Figure 7:**
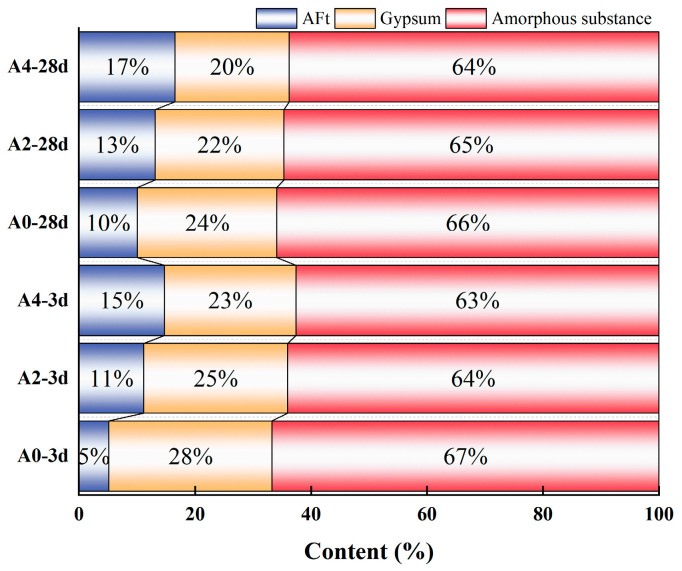
Phase content of different samples.

The determination of gypsum, ettringite, and amorphous material content in the samples evaluated by the internal standard method is shown in Figure 7. With increasing sodium aluminate content, all samples exhibit a consistent trend of rising ettringite formation accompanied by decreasing gypsum content. The addition of sodium aluminate can accelerate the early hydration reaction rate and increase the amount of ettringite in the early hydration stage. At 3 d, the ettringite content in the A0 group (without sodium aluminate) accounts for only 5 wt.% of the total substance content. In contrast, the ettringite content in the A2 group increases to 11 wt.%, more than double that of the A0 group. The A4 group exhibited a higher ettringite content of 15 wt.%, which is three times that of the A0 group. At 28 d, the ettringite content of the A4 group reached 17 wt.%, which is 7 wt.% higher than that of the A0 group.

At the same time, the addition of sodium aluminate accelerates the decomposition of phosphogypsum and the early hydration reaction speed, thereby reducing the residual amount of gypsum in the samples. After 3 d of hydration, the gypsum content in the A4 group is 23 wt.%, which was 5 wt.% lower than that in the A0 group. After 28 d of hydration, the phosphogypsum content of the A4 group decreases to 20 wt.%, which is 4 wt.% lower than that of the A0 group. As the age of the hydration reaction increases, the gypsum content also shows a progressive decrease. The gypsum content of the A0 group decreases from 28 wt.% to 24 wt.%, while that of the A4 group decreases from 23 wt.% to 20 wt.%. The residual amount of phosphogypsum significantly affects the mechanical properties and durability of the material. Further, a lower residual amount of phosphogypsum in PBCMs is beneficial for durability in later age [27].

The amorphous substance contains un-hydrated GGBS particles and C-(A)-S-H gel. The addition of sodium aluminate accelerates the dissolution of GGBS and promotes the formation of more amorphous gel. At the same age of hydration reaction, with the increase in sodium aluminate content, the amorphous material content shows a decreasing trend. At 3 d, the content of the amorphous substance of A0 is 67 wt.%, while that of A2 and A4 is 64 wt.% and 63 wt.%. However, as hydration progresses from 3 d to 28 d, the amorphous material content of A0, A2, and A4 increased by 1%, respectively, which is because the generation of the amorphous gel of the hydration product is larger than the consumption of the GGBS content.

In general, the addition of sodium aluminate can accelerate the decomposition of phosphogypsum and GGBS, prompting greater participation in the hydration reaction, thus accelerating the rate of the early hydration reaction and promoting the formation of ettringite and amorphous gel substances such as C-(A)-S-H gel.

### 3.5. SEM

**Figure 8:**
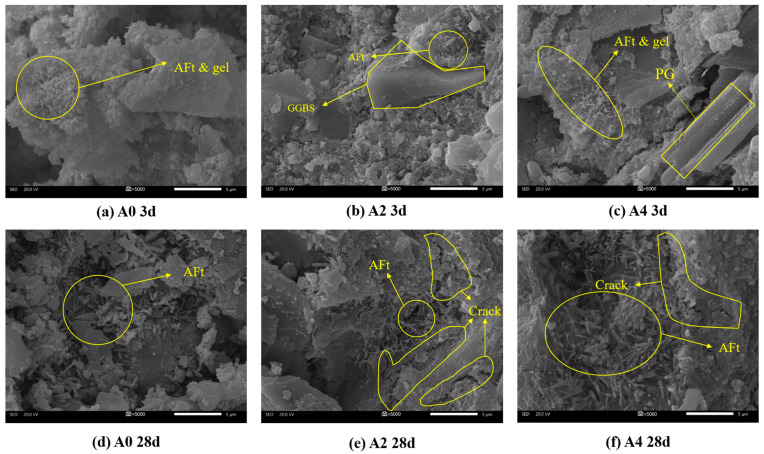
SEM images of PBCM with different sodium aluminate contents at 3 d and 28 d.

Figure 8 presents the microscopic morphology of samples A0, A2, and A4 hydrated at 3 d and 28 d, respectively. Figure 8a shows the SEM image of the 3 d hydration of group A0 without sodium meta-aluminate. In this sample, the ettringite (AFt) crystals are few in number and small in size. These crystals are distributed on the surface of GGBS particles, with some gel surrounding them [41]. However, the ettringite does not form a continuous skeletal structure through interconnection, resulting in a sparse microstructure. This contributes to the low 3 d compressive strength observed in group A0.

As hydration progresses from 3 d to 28 d, both the crystal size and quantity of ettringite increases significantly in all three groups. It can be seen that the amount of ettringite in the 28 d samples is greater than that in the 3 d samples. The adjacent ettringite crystals begin to overlap and interlock, forming a skeleton that helps to fill the pores between particles, thereby enhancing the overall compressive strength [42,43,44].

By comparing Figure 8a–c, it is evident that the A0 group exhibits fewer and smaller ettringite crystals at 3 d. In contrast, the A2 and A4 groups show a notable increase in both the number and size of ettringite crystals, resulting in a denser microstructure. The increase in sodium aluminate content leads to a significant rise in ettringite formation, which enhances the compressive strength. The results support the analytical findings discussed in Section 3.1.

Although the addition of sodium aluminate can accelerate the early-stage hydration reaction, a large amount of the hydration products generated at early ages tend to wrap around the aluminosilicate minerals, thereby hindering further hydration. In addition, the rapidly formed hydration products induce the formation of numerous microcracks, as observed in Figure 8b,c.

As curing age progresses beyond 7 d, the increase in ettringite content (discussed in Section 3.4) intensifies volumetric expansion, leading to a greater number of microcracks as shown in Figure 8e. Additionally, the incomplete encapsulation of ettringite crystals by C-(A)-S-H gel facilitates microcrack propagation. This is why the compressive strength of Groups A1, A2, and A3 at 28 d is lower than that at 7 d. But for Group A4, more aluminum hydroxide gel fills the cracks and make the structure more stable in the early stage. As the curing age increases, it reacts with sulfate and calcium ions to form ettringite and C-A-S-H gel, which compensates for the loss of strength and structural defects caused by cracks [45].

As shown in Figure 8b, the hydration products generated during the reaction tend to encapsulate unreacted gypsum and GGBS particles. Sodium aluminate accelerates early hydration, resulting in the rapid formation of hydration products that accumulate on the GGBS surface and encapsulate the particles. This encapsulation hinders the dissolution of active slag components, thereby suppressing further strength development [30,46,47]. Consequently, the 28 d strength of A1, A2, and A3 is lower than that of the A0 group.

### 3.6. Mechaisim Analysis

When quicklime is mixed with water, it reacts to form calcium hydroxide, which dissociates into calcium ions and hydroxide ions in the paste, as shown in Equation (1). Sodium aluminate combines with water to form tetrahydroxy aluminate as in Equation (2), which consumes a portion of water, reducing the effective water available in the system [48,49]. In addition, it reacts with calcium hydroxide to form substantial quantities of aluminum hydroxide gel and calcium aluminate precipitates as shown in Equation (3) [50], thereby increasing the viscosity and leading to a thicker paste. Further, the by-product, sodium hydroxide, further increases the alkalinity of the paste, reducing the solubility of polycarboxylate superplasticizer (PCE). And the main chain of PCE coils, weakening its steric hindrance, and tetrahydroxy aluminate anions compete with PCE for adsorption sites on the slag surface, thus diminishing the adsorption effect [51,52]. As a result, the water-reducing effect of the superplasticizer diminishes, resulting in a significant reduction in fluidity.

During the early hydration process of PBCMs, abundant sulfate and calcium ions are supplied by phosphogypsum in the slurry. The formation rate of aluminum octahedra is the rate-controlling step in the formation of ettringite [53]. However, the dissolution of the active aluminum components in GGBS is relatively slow, which constrains the development of strength and the formation of hydration products. After the addition of sodium aluminate, it acts as the active aluminum component in the system, becoming the main aluminum source during the early hydration reaction. In the alkaline environment, sodium aluminate accelerates the formation of aluminum octahedra, promoting the forward progress of Equation (4). This leads to the rapid combination of aluminate ions with sulfate and calcium ions, resulting in the formation of ettringite and accelerating its nucleation and growth [54], as shown in Equation (5). The rapidly generated hydration products shorten the setting time of the cementitious material and significantly enhance its early strength [31,55], as shown in Reaction (6). Furthermore, sodium aluminate promotes the further dissolution of the active components in GGBS, facilitating the formation of C-A-S-H and N-A-S-H gels [34,56,57], as described in Equations (6) and (7).

(1) $$CaO+H_{2}O\to Ca{\left(OH\right)}_{2}$$

(2) $$NaAlO_{2}+H_{2}O\to NaAl{\left(OH\right)}_{4}$$

(3) $$NaAl{\left(OH\right)}_{4}+Ca{\left(OH\right)}_{2}\to Al{\left(OH\right)}_{3}↓+{Ca\left(AlO_{2}\right)}_{2}↓+NaOH+H_{2}O$$

(4) $$Al{\left(OH\right)}_{4}^{-}+2{OH}^{-}\to Al{\left(OH\right)}_{6}^{3-}$$

(5) $$Al{\left(OH\right)}_{6}^{3-}+6{Ca}^{2+}+{3SO}_{4}^{2-}+{OH}^{-}+2H_{2}O\to AFt$$

(6) $$Ca^{2+}+{\;[\mathrm{Al}O_{4}]}^{4-}+{\;[\mathrm{Si}O_{4}]}^{4-}+{OH}^{-}+H_{2}O\to C-A-S-H$$

(7) $$Na^{+}+{\;[\mathrm{Al}O_{4}]}^{4-}+{\;[\mathrm{Si}O_{4}]}^{4-}+{OH}^{-}+H_{2}O\to N-A-S-H$$

## 4. Conclusions

In this study, the effect of sodium aluminate addition on the early performance of low-carbon cementitious materials was investigated. The PBCMs consisted of 98 wt.% solid waste including a high phosphogypsum content (45 wt.%). The results reveal that this could significantly improve the early performance. The conclusions are as follows:

(1) Sodium aluminate significantly enhances the 3 d strength of PBCMs. As the sodium aluminate content increases, the 3 d strength of PBCMs improves. When the content of sodium aluminate is 1 wt.%, the 3 d compressive strength of PBCMs reaches 10.72 MPa, a 587.39% increase compared to A0. And the 7 d strength of A4 reaches 15.57 MPa, a 39.66% improvement, while the 28 d compressive strength is nearly identical to that of the A0 group.
(2) The addition of sodium aluminate negatively affected the fluidity of the slurry. When the content reached 0.8%, the slurry lost its fluidity.
(3) The addition of sodium aluminate significantly shortens the setting time of PBCM. As the content of sodium aluminate increases, the setting time is progressively shortened. When the contents of sodium aluminate are 0.8 wt.% and 1 wt.%, the initial setting time is reduced by 4 h 15 min and 4 h 24 min, corresponding to final setting time reductions of approximately 3 h 53 min and 4 h 4 min compared to the control group.
(4) Sodium aluminate effectively promotes the decomposition of phosphogypsum and accelerates the early hydration reaction, enhancing the formation of early hydration products, improving early strength, and shortening the setting time.

## Figures and Tables

**Table 1:** Chemical composition of PG and GGBS (wt.%).

	SiO_2_	Al_2_O_3_	SO_3_	CaO	Fe_2_O_3_	P_2_O_5_	MgO	Na_2_O	K_2_O	BaO
PG	5.6	0.363	51.88	40.78	0.277	0.707	0.062	-	0.0883	0.12
GGBS	30.99	12.30	2.23	33.8	1.01	0.031	7.96	0.586	0.725	0.15

**Table 2:** Mix proportion design (wt.%).

No.	Phosphogypsum	Slag	Lime	Sodium Aluminate	Superplasticizer
A0	45	53	2	0.00	0.05
A1	45	53	2	0.20	0.05
A2	45	53	2	0.50	0.05
A3	45	53	2	0.80	0.05
A4	45	53	2	1.00	0.05

## Data Availability

The original contributions presented in this study are included in the article. Further inquiries can be directed to the corresponding author.

## References

[1] Chen Q.S., Zhang Q.L., Qi C.C., Fourie A., Xiao C.C. (2018). Recycling phosphogypsum and construction demolition waste for cemented paste backfill and its environmental impact. J. Clean. Prod..

[2] Chernysh Y., Yakhnenko O., Chubur V., Roubík H. (2021). Phosphogypsum Recycling: A Review of Environmental Issues, Current Trends, and Prospects. Appl. Sci..

[3] Pérez-López R., Macías F., Cánovas C.R., Sarmiento A.M., Pérez-Moreno S.M. (2016). Pollutant flows from a phosphogypsum disposal area to an estuarine environment: An insight from geochemical signatures. Sci. Total Environ..

[4] Arhouni F.E., Hakkar M., Ouakkas S., Haneklaus N., Boukhair A., Nourreddine A., Benjelloun M. (2023). Evaluation of the physicochemical, heavy metal and radiological contamination from phosphogypsum discharges of the phosphoric acid production unit on the coast of El Jadida Province in Morocco. J. Radioanal. Nucl. Chem..

[5] Ren K., Cui N., Zhao S.Y., Zheng K., Ji X., Feng L.C., Cheng X., Xie N. (2021). Low-Carbon Sustainable Composites from Waste Phosphogypsum and Their Environmental Impacts. Crystals.

[6] Wang Z.Y., Ma X.H., Pan H.Y., Yang X.D., Zhang X.H., Lyu Y., Liao W.J., Shui W., Wu J., Xu M. (2023). Investigating effects of phosphogypsum disposal practices on the environmental performance of phosphate fertilizer production using emergy analysis and carbon emission amounting: A case study from China. J. Clean. Prod..

[7] Duan Z.Y., Li J.X., Li T.G., Zheng S.R., Han W.M., Geng Q.Y., Guo H.B. (2017). Influence of crystal modifier on the preparation of α-hemihydrate gypsum from phosphogypsum. Constr. Build. Mater..

[8] Kadirova Z.C., Hojamberdiev M., Bo L.L., Hojiyev R., Okada K. (2014). Ion uptake properties of low-cost inorganic sorption materials in the CaO-Al2O3-SiO2 system prepared from phosphogypsum and kaolin. J. Clean. Prod..

[9] Rashad A.M. (2017). Phosphogypsum as a construction material. J. Clean. Prod..

[10] Xu J., Xu F., Jiang Y., Jiao Y.Y., Sun T., Yang F., Wu Y., Chen S.Y., Liu Y.M., Zhu J. (2023). Mechanical properties and soluble phosphorus solidification mechanism of a novel high amount phosphogypsum-based mortar. Constr. Build. Mater..

[11] Yang L., Zhang Y.S., Yan Y. (2016). Utilization of original phosphogypsum as raw material for the preparation of self-leveling mortar. J. Clean. Prod..

[12] Lin Z.S., Zhao Q. (2009). Strength of limestone-based non-calcined cement and its properties. J. Wuhan Univ. Technol. Mater. Sci. Ed..

[13] Huang Y., Lin Z.S. (2010). Investigation on phosphogypsum-steel slag-granulated blast-furnace slag-limestone cement. Constr. Build. Mater..

[14] Amrani M., Taha Y., Kchikach A., Benzaazoua M., Hakkou R. (2020). Phosphogypsum recycling: New horizons for a more sustainable road material application. J. Build. Eng..

[15] Lam N.N. A study on super-sulfated cement using Dinh Vu phosphogypsum. Proceedings of the 2nd International Conference on Sustainable Development in Civil, Urban and Transportation Engineering, CUTE 2018.

[16] Chen M.S., Liu P., Kong D.W., Wang Y., Wang J.D., Huang Y.S., Yu K., Wu N.B. (2022). Influencing factors of mechanical and thermal conductivity of foamed phosphogypsum-based composite cementitious materials. Constr. Build. Mater..

[17] Liu X.M., Liu E. (2023). The Synergistic Mechanism and Stability Evaluation of Phosphogypsum and Recycled Fine Powder-Based Multi-Source Solid Waste Geopolymer. Polymers.

[18] Liu X.M., Liu E.R., Fu Y.T. (2023). Reduction in Drying Shrinkage and Efflorescence of Recycled Brick and Concrete Fine Powder-Slag-Based Geopolymer. Appl. Sci..

[19] Liu X.M., Li T.Y., Tian W.G., Wang Y.Q., Chen Y.H. (2020). Study on the durability of concrete with FNS fine aggregate. J. Hazard. Mater..

[20] Zhang L.J., Mo K.H., Yap S.P., Gencel O., Ling T.C. (2022). Mechanical strength, water resistance and drying shrinkage of lightweight hemihydrate phosphogypsum-cement composite with ground granulated blast furnace slag and recycled waste glass. Constr. Build. Mater..

[21] Dong M., Li J.S., Lang L., Chen X., Jin J.X., Ma W. (2023). Recycling thermal modified phosphogypsum in calcium sulfoaluminate cement: Evolution of engineering properties and micro-mechanism. Constr. Build. Mater..

[22] Sun B.B., Wu H., Song W.M., Li Z., Yu J. (2020). Hydration, microstructure and autogenous shrinkage behaviors of cement mortars by addition of superabsorbent polymers. Front. Struct. Civ. Eng..

[23] Pinto S.R., da Luz C.A., Munhoz G.S., Medeiros R.A. (2020). Durability of phosphogypsum-based supersulfated cement mortar against external attack by sodium and magnesium sulfate. Cem. Concr. Res..

[24] Pratap B., Mondal S., Rao B.H. (2024). Mechanical and durability assessment of phosphogypsum-bauxite residue-fly ash-based alkali-activated concrete. Constr. Build. Mater..

[25] Wang Z.Y., Shui Z.H., Sun T., Li Z.W. (2022). An Eco-Friendly Phosphogypsum-Based Cementitious Material: Performance Optimization and Enhancing Mechanisms. Front. Phys..

[26] Wang Z.Y., Shui Z.H., Sun T., Li X.S., Zhang M.Z. (2022). Recycling utilization of phosphogypsum in eco excess-sulphate cement: Synergistic effects of metakaolin and slag additives on hydration, strength and microstructure. J. Clean. Prod..

[27] Zhao B., Wang G.J., Zhao K., Wang M.L., Wu B.S., Li S.J., Chen Q.L., Geng J.B. (2023). Mechanical properties, permeability and microstructure of steam-cured fly ash mortar mixed with phosphogypsum. Constr. Build. Mater..

[28] Luo Y., Ma B., Liang F., Xue Z., Qian B., Wang J., Zhou L., Zang J., Liang R., Li Y. (2023). Use of untreated phosphogypsum as a raw material for autoclaved aerated concrete preparation. J. Build. Eng..

[29] Li W.X., Xie Y.J., Ma K.L., Long G.C., Zhao H., Peng Y.M. (2023). Multiscale mechanical evolution of the interface between self-compacting concrete and steam-cured concrete. J. Build. Eng..

[30] Lin H., Li Y., Yang B., Xie D. (2024). Effects of different admixtures on the working performance and mechanical properties of phosphogypsum slag-based composite cementitious materials. J. Build. Eng..

[31] Yang R.H., He T.S. (2022). Influence of liquid accelerator based on sodium aluminate on the early hydration of cement, C3A and C3S pastes. Adv. Cem. Res..

[32] Lv Q.F., Wang Z.S., Gu L.Y., Chen Y., Shan X.K. (2020). Effect of sodium sulfate on strength and microstructure of alkali-activated fly ash based geopolymer. J. Cent. South Univ..

[33] Wang Y.L., Yu J., Wang J.J., Xiang D., Gu H., Cheng J.H. (2022). Effects of sodium aluminate and quicklime on the properties of CSA grouting materials. J. Build. Eng..

[34] Han J.G., Wang K.J., Shi J.Y., Wang Y. (2014). Influence of sodium aluminate on cement hydration and concrete properties. Constr. Build. Mater..

[35] (2024). Methods for Determining Water Requirement for Standard Consistency, Setting Time, and Stability of Cement.

[36] (2023). Test Methods for the Homogeneity of Concrete Admixtures.

[37] Tang P., Wen J., Fu Y., Liu X., Chen W. (2024). Improving the early-age properties of eco-binder with high volume waste gypsum: Hydration process and ettringite formation. J. Build. Eng..

[38] Song P.F., Wang X.H., Wang Y., Zhou J., Qiu H.P., Rahimi A., Ingham J. (2025). Assess the interaction of water reducers and accelerators on the rheological and early hydration properties of cement-based materials. J. Mater. Res. Technol..

[39] Ooi W.E., Liew Y.M., Heah C.Y., Abdullah M.M.A., Li L.Y., Ho L., Loong F.K., Shee-Ween O., Ng H.T., Ng Y.S. (2021). Comparative mechanical and microstructural properties of high calcium fly ash one-part geopolymers activated with Na2SiO3-anhydrous and NaAlO_2_. J. Mater. Res. Technol..

[40] Chen B., Wang J., Zhao J.Y. (2021). Effect of Sodium Aluminate Dosage as a Solid Alkaline Activator on the Properties of Alkali-Activated Slag Paste. Adv. Mater. Sci. Eng..

[41] Dan H.C., Ma Z.M., Li M.J., Ma S.L., Tan J.W. (2023). Early performance and bonding mechanism of metakaolin (MK)- ground granulated blast furnace slag (GGBS) based geopolymer road repair mortar. Int. J. Pavement Eng..

[42] Jiang D.B., Li X.G., Jiang W.G., Li C.J., Lv Y., Zhou Y. (2020). Effect of tricalcium aluminate and sodium aluminate on thaumasite formation in cement paste. Constr. Build. Mater..

[43] Liu J., Ma K.L., Shen J.T., Zhu J.B., Long G.C., Xie Y.J., Liu B.J. (2023). Influence of recycled concrete aggregate enhancement methods on the change of microstructure of ITZs in recycled aggregate concrete. Constr. Build. Mater..

[44] Song W.M., Zhang M.M., Wu H. (2024). Gray correlation analysis between mechanical performance and pore characteristics of permeable concrete. J. Build. Eng..

[45] Ma K.L., Feng K.W., Wang Z.Z., Long G.C., Xie Y.J., Li W.X. (2023). Mechanical properties and crack evolution of SCC with macro-crack under freeze-thaw cycles. J. Build. Eng..

[46] Peng Y.M., Ma K.L., Long G.C., Xie Y.J., Yu L.N., Xie Q.Q. (2021). Effect of Packing Density According to CPM on the Rheology of Cement-Fly Ash-Slag Paste. J. Mater. Civ. Eng..

[47] Meng W.Q., Ma K.L., Long G.C., Meng Q.Y., Chen R.J., Fan J.Z. (2024). Influence of Molybdenum Tailings Powder on the Hydration Characteristics of Cement Paste. J. Mater. Civ. Eng..

[48] Wang J.H., Xie Y.J., Zhong X.H., Li L.J. (2020). Test and simulation of cement hydration degree for shotcrete with alkaline and alkali-free accelerators. Cem. Concr. Compos..

[49] Wu H., Li Z., Song W.M., Bai S. (2021). Effects of superabsorbent polymers on moisture migration and accumulation behaviors in soil. J. Clean. Prod..

[50] Zhang B.Y., Pan X.L., Wang J.Z., Yu H.Y., Tu G.F. (2019). Reaction kinetics and mechanism of calcium oxide in dilute sodium aluminate solution with oxalate based on lime causticization. Trans. Nonferrous Met. Soc. China.

[51] Chen J.X., Plank J. (2025). Which factors impact the effectiveness of PCEs in alkali-activated slag cements?. Cem. Concr. Res..

[52] Feng K.W., Ma K.L., Yang H.Z., Long G.C., Xie Y.J., Zeng X.H., Tang Z., Usman I.U. (2024). Influence of cellulose ethers on rheological properties of cementitious materials: A review. J. Build. Eng..

[53] Peng J.H., Zhang J.X., Qu J.D. (2006). The mechanism of the formation and transformation of ettringite. J. Wuhan Univ. Technol. Mater. Sci. Ed..

[54] Herrera-Mesen C., Salvador R.P., Ikumi T., Cavalaro S.H.P., Aguado A. (2020). External sulphate attack of sprayed mortars with sulphate-resisting cement: Influence of accelerator and age of exposition. Cem. Concr. Compos..

[55] Liu Y.T., Zhang Y.Y., Dong B.Q., Wang Y.S. (2023). Limestone powder activated by sodium aluminate: Hydration and microstructure. Constr. Build. Mater..

[56] Ma C., Zhao B., He Y.Z., Li F., Long G.C., Du Y.F. (2020). Preparation and properties of sustainable magnesium phosphate cement composites with recycled tire rubber particles. J. Clean. Prod..

[57] Song W.M., Yi J., Wu H., He X., Song Q.W., Yin J. (2019). Effect of carbon fiber on mechanical properties and dimensional stability of concrete incorporated with granulated-blast furnace slag. J. Clean. Prod..

